# Application of kt-BLAST acceleration to reduce cardiac MR imaging time in healthy and infarcted mice

**DOI:** 10.1007/s10334-013-0392-5

**Published:** 2013-07-09

**Authors:** Ian Marshall, Maurits A. Jansen, Yuehui Tao, Gavin D. Merrifield, Gillian A. Gray

**Affiliations:** 1 Centre for Clinical Brain Sciences, University of Edinburgh, Edinburgh, UK; 2 University/British Heart Foundation Centre for Cardiovascular Science, University of Edinburgh, Edinburgh, UK; 3 Department of Cardiovascular Medicine, University of Oxford, Oxford, UK; 4 SINAPSE Consortium, Edinburgh, UK

**Keywords:** Mouse, Cardiac, Acceleration, Cine, Myocardial infarction

## Abstract

**Object:**

We evaluated the use of kt-broad-use linear acquisition speed-up technique (kt-BLAST) acceleration of mouse cardiac imaging in order to reduce scan times, thereby minimising physiological variation and improving animal welfare.

**Materials and methods:**

Conventional cine cardiac MRI data acquired from healthy mice (*n* = 9) were subsampled to simulate kt-BLAST acceleration. Cardiological indices (left ventricular volume, ejection fraction and mass) were determined as a function of acceleration factor. kt-BLAST threefold undersampling was implemented on the scanner and applied to a second group of mice (*n* = 6 healthy plus 6 with myocardial infarct), being compared with standard cine imaging (3 signal averages) and cine imaging with one signal average.

**Results:**

In the simulations, sufficient accuracy was achieved for undersampling factors up to three. Cardiological indices determined from the implemented kt-BLAST scanning showed no significant differences compared with the values determined from the standard sequence, and neither did indices derived from the cine scan with only one signal average despite its lower signal-to-noise ratio. Both techniques were applied successfully in the infarcted hearts.

**Conclusion:**

For cardiac imaging of mice, threefold undersampling of kt-space, or a similar reduction in the number of signal averages, are both feasible with subsequent reduction in imaging time.

## Introduction

In recent years there has been a significant increase in in vivo imaging studies of experimental rodents. This has been driven by several factors, including the desirability of non-invasive, longitudinal examinations of disease progression, the availability of genetically modified animals, and technological developments enabling the translation of clinical imaging techniques to the animal laboratory. Magnetic resonance imaging (MRI) is particularly versatile, providing anatomical, functional, and dynamic information, and is now widely established for the imaging of rats and mice [1], including cardiac studies [2–5]. The main drawback to MRI is the examination time, which is typically 1 h or more per animal. Such prolonged periods of anaesthesia are potentially harmful to small animals, especially when imaged shortly after surgical intervention (e.g., for induction of myocardial infarct), requiring careful monitoring during subsequent recovery periods. Shorter scan times would improve animal welfare by reducing this exposure to anaesthesia, would minimise the effects of physiological instability during scanning, and would also reduce study costs.

MRI can be accelerated by acquiring less than the conventional number of rows of k-space. Two different approaches are possible. In so-called “parallel imaging”, ambiguities of reconstruction are resolved by using prior information on the spatial sensitivity patterns of multiple receiver coils [6, 7]. In a second class of techniques, particularly applicable to dynamic scanning, undersampling of kt-space exploits spatiotemporal correlations in periodic data [8–11] and does not require special receiver coils. In a third class of techniques, Wech et al. [12] recently demonstrated threefold acceleration of mouse cardiac imaging using a compressed sensing [13] approach to exploit sparsity in the raw data.

The various techniques have achieved accelerations of typically two to four times, and may be combined [9]. Parallel imaging using phased array receiver coils is now widely used to reduce scan times in clinical MRI, whereas dynamic undersampling is less well established. There have been very few reports of translating the undersampling techniques to the imaging of experimental animals. The additional challenges of small animal imaging include limited signal-to-noise ratio (SNR) and the difficulty of designing non-interacting RF coils for parallel acquisition at the high frequencies encountered in typical preclinical scanners (200 MHz and above). Despite these difficulties, parallel imaging in rat [14] and mouse [15] cardiac studies have recently been demonstrated by Schneider et al. Ratering et al. [16] have also demonstrated parallel imaging to accelerate MR angiography in mice.

In this work, we explored the feasibility of the kt-broad-use linear acquisition speed-up technique (kt-BLAST) [9] acceleration of mouse cardiac imaging. We first subsampled existing full k-space datasets to determine a suitable acceleration factor, and then implemented accelerated scanning on a small animal scanner. Conventional and accelerated cardiac images were compared qualitatively and by determination of standard cardiological indices in both healthy and myocardial infarcted animals.

## Materials and methods

### Simulated undersampling

All experiments conformed to national and institutional regulations for animal care. Male 10–12 week old C57BL/6 mice (*n* = 9) were anaesthetised with isoflurane at 1.5 ± 0.3 % to maintain respiration at 50–60 breaths per minute as monitored by a pressure pad. Heart-rate was monitored from ECG recorded using needle electrodes inserted subdermally on either side of the heart (Small Animal Instruments Inc., Stony Brook, USA). Rectal temperature was maintained at 37 °C by a hot air blower with feedback control. Animals were scanned using a 7T MRI system (Agilent Technologies, formerly Varian Inc.) with a 39-mm quadrature coil. Prospective cardiac synchronisation and respiratory gating were used. The cardiac short-axis was identified from a series of localiser scans broadly as described by Schneider et al. [3]. Short-axis cardiac images were acquired with a gradient echo ‘cine’ sequence (TR/TE = 7.3/2.7 ms, flip angle 15°) with gradient and RF spoiling, consisting of 12–13 time frames, 9 contiguous slices of 1 mm thickness, FOV 30 mm, a 192 × 192 matrix and 4 averages. Raw k-space data were processed using custom software. kt-BLAST simulations written in Matlab consisted of selecting rows of k-space from 12 time frames to represent “training” data (central 16 rows) and “*r*-fold accelerated acquisition” data (every *r*th row of k-space, where *r* = [2–4, 6] and the index of the first row is incremented between time frames). Training and accelerated data were used to reconstruct image sets using the standard kt-BLAST algorithm [9]. The magnitude “reconstruction error” *ε* between reconstruction from full k-space and from undersampled k-space, normalised to the values from the full reconstruction, was calculated for a 60 × 60 pixel region of interest (ROI) placed around the heart and averaged over all slices, time frames and pixels for a given animal and acceleration. Mathematically,

1 $$ \varepsilon = \frac{1}{nt \times ns \times nx \times ny}\sum {\frac{{\left| {I_{f} - I_{u} } \right|}}{{I_{f} }}} $$

where *nt*, *ns*, *nx* and *ny* are respectively the numbers of time frames, slices, pixels in the *x*-direction and pixels in the *y*-direction within the ROI. *I*_*f*_ refers to the image reconstructed from full k-space and *I*_*u*_ that from undersampled k-space. The summation is over all *t*, *s*, *x* and *y*. This ROI size was chosen to capture ghosting artefacts from undersampling which are expected to appear both inside and outside the heart. Cardiological indices of left ventricular function (end diastolic volume EDV, end systolic volume ESV, stroke volume SV, ejection fraction EF, end diastolic mass EDM, end systolic mass ESM) were measured from all image sets by one experienced analyst who was blinded to the identity of each set. End systolic and end diastolic frames were selected visually. Measurements were made by drawing around epicardial and endocardial boundaries in ImageJ software (http://rsbweb.nih.gov/ij). The papillary muscles were excluded from the LV blood pool, and a density of 1.05 g/cm^3^ was assumed for the myocardium. A subjective rating of quality was also given to each set, ranging from 1 (“poor”) through to 5 (“good”). This subjective rating was based on a combination of apparent SNR, severity of artefacts, delineation of the myocardium and ease of measurement. After unblinding, two-tailed *t* tests were used to determine whether the cardiological indices measured at each acceleration factor were significantly different (*p* < 0.05) from those measured using full k-space reconstruction.

### Accelerated acquisition

A suitable acceleration factor of three was determined from the simulated undersampling experiments (see “Results” section). kt-BLAST undersampling with this acceleration factor was implemented on the scanner, together with a training sequence that acquired the central 16 rows of k-space. A further 6 mice (body weights 28–55 g) were each imaged with (i) the standard cine sequence as above, except that the heart was covered in 8 slices with three signal averages being acquired (CINE-3); (ii) the kt-BLAST undersampling and training sequences as implemented (ktB-3), each with three signal averages; (iii) the standard sequence but with only one signal average (CINE-1). All other imaging parameters were kept the same. In order to evaluate the accelerated sequence with pathology, 6 mice in which myocardial infarct had been induced by permanent ligation of the left anterior descending coronary artery [17] were scanned with this protocol (five only with the CINE-1 sequence) between 4 and 7 days after induction of infarct. The infarcted mice (body weights 22–30 g) were scanned using a 33 mm coil. The three scanning sequences were run in the stated order for all animals. Acquisition time was approximately 20 min for the full cine sequence, and 7 min each for the undersampling sequence and cine sequence with one average. A further 2 min was required for the training sequence. All image sets were reconstructed, scored qualitatively, and the cardiological indices measured as above, with the analyst again being blinded to their identity. After unblinding, the cardiological indices for each animal measured with the accelerated scan and the cine scan with one average were compared using Bland–Altman analysis with those obtained from the standard full cine scan with three averages. Two-tailed *t* tests were used to determine whether Bland–Altman biases were significant.

A similar comparison was also made between the accelerated and single-average images.

The contrast to noise ratio (CNR) between the left ventricular blood pool and the myocardium was determined using the relationship CNR = (*S*_blood_ − *S*_myo_)/*σ* where *S*_blood_ is the mean signal level in the left ventricle, *S*_myo_ is the mean signal level in the septum, and *σ* is the combined (square root of sum of squares) standard deviation of *S*_myo_ and *S*_blood_, all measured on a mid-ventricular slice at end diastole.

## Results

### Simulated undersampling

**Fig. 1:**
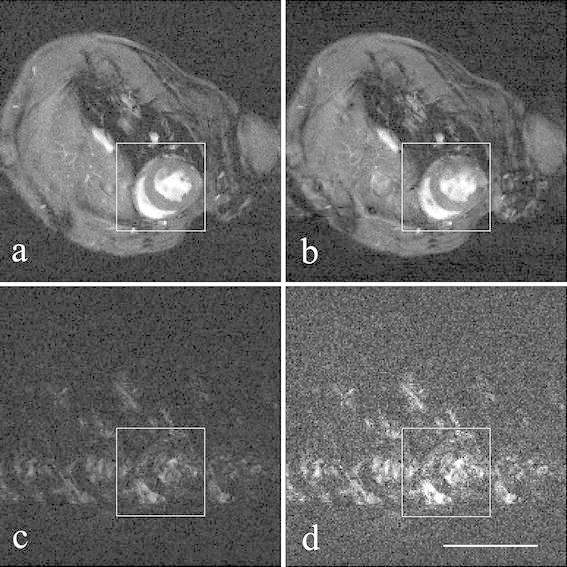
Representative mouse cardiac images at end diastole reconstructed using full k-space data (**a**) and simulated fourfold kt-BLAST undersampling (**b**); magnitude difference image between (**a**) and (**b**), displayed with the same (**c**) and with threefold expanded windowing (**d**) to emphasise the differences. The ROI for error analysis is indicated. In this example, the calculated reconstruction error *ε* was 0.53. The image quality was judged “good” for the full reconstruction and “reasonable” for the fourfold undersampled reconstruction. Each image has a FOV of 30 mm. *Scale bar* 10 mm

**Fig. 2:**
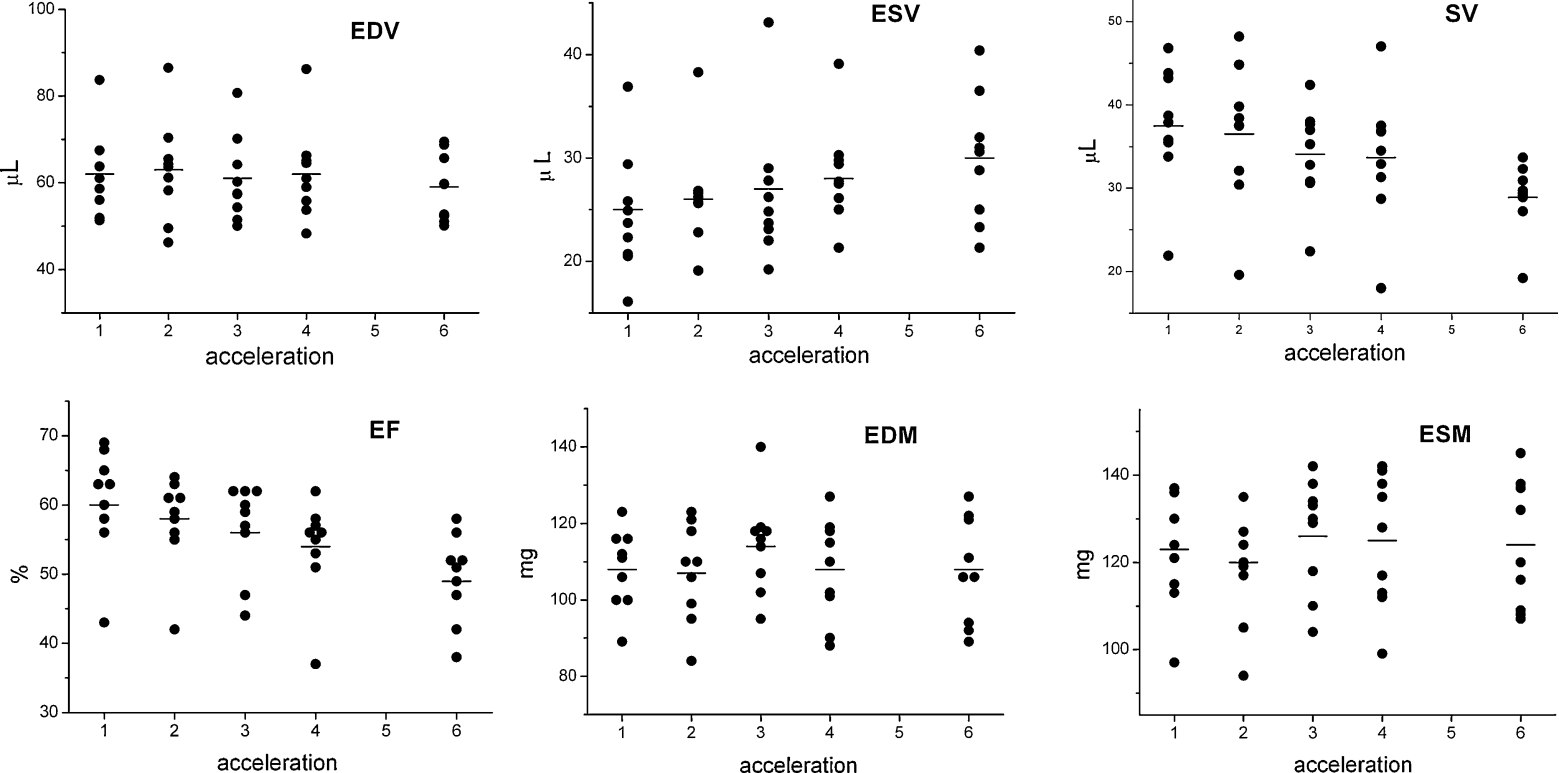
Cardiological indices of left ventricular function in healthy mice (*n* = 9) measured from images with simulated acceleration, plotted against acceleration factor. *EDV* end diastolic volume, *ESV* end systolic volume, *SV* stroke volume, *EF* ejection fraction, *EDM* end diastolic mass, *ESM* end systolic mass. *Horizontal lines* indicate the group means. See also Table 1

Figure 1 shows representative mid-ventricular images at early systole from the simulated acceleration study. The reconstruction error *ε*, averaged over all animals (*n* = 9), increased, and the subjective quality rating decreased as the simulated acceleration factor *r* increased (Table 1). The effect of simulated acceleration on the measured cardiological parameters is shown in Table 1 and Fig. 2. Notably, as the acceleration factor was increased, measured ESV increased whilst measured EDV was approximately maintained. Consequently, the estimated stroke volume and ejection fraction fell from their “true” values measured from full k-space data, with the difference reaching statistical significance for *r* = 6. Estimates of left ventricular mass were not significantly altered by simulated undersampling.

**Table 1:** Simulated kt-BLAST acceleration

Acceleration factor	Error *ε*	Quality (Q)	EDV (μL)	ESV (μL)	SV (μL)	EF (%)	EDM (mg)	ESM (mg)
1 (full k-space)	0.00	4.2 ± 1.1	62 ± 10	25 ± 6	38 ± 7	60 ± 8	108 ± 10	123 ± 13
2	0.32 ± 0.06	3.7 ± 1.1	63 ± 12	26 ± 5	36 ± 8	58 ± 6	107 ± 13	120 ± 13
3	0.39 ± 0.06	3.1 ± 0.6*	61 ± 10	27 ± 7	34 ± 6	56 ± 7	114 ± 13	126 ± 13
4	0.45 ± 0.07	2.4 ± 1.1*	62 ± 11	28 ± 5	34 ± 8	54 ± 7	108 ± 13	125 ± 15
6	0.55 ± 0.08	1.1 ± 0.3*	59 ± 8	30 ± 6	29 ± 4*	49 ± 6*	108 ± 14	124 ± 15

Reconstruction error (*ε*: see text), subjective image quality rating (Q), and cardiological indices of left ventricular function (mean ± standard deviation) in healthy mice (*n* = 9) measured from images with simulated kt-BLAST acceleration. The range of Q is from 1 (“poor”) through to 5 (“good”). *EDV* end diastolic volume, *ESV* end systolic volume, *SV* stroke volume, *EF* ejection fraction, *EDM* end diastolic mass, *ESM* end systolic mass. * Significantly different (*p* < 0.05) from the full k–space values. See also Fig. 2

### Accelerated acquisition

**Fig. 3:**
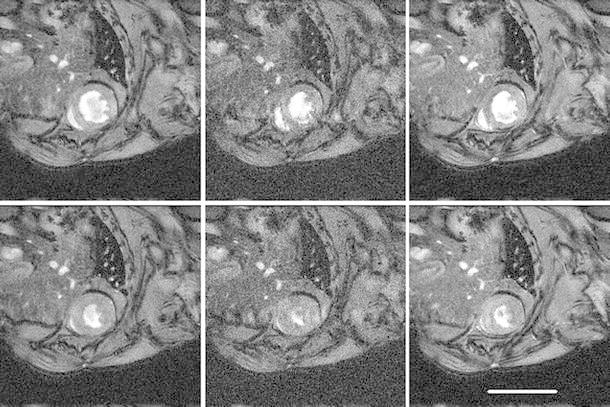
Representative cardiac images of a healthy mouse. *Upper row* acquired at end diastole and *lower row* at end systole. *Left* to *right* standard cine sequence with three signal averages (CINE-3); cine sequence with one signal average (CINE-1); kt-BLAST reconstruction of a threefold undersampled sequence (ktB-3). Each image has a FOV of 30 mm. *Scale bar* 10 mm

**Fig. 4:**
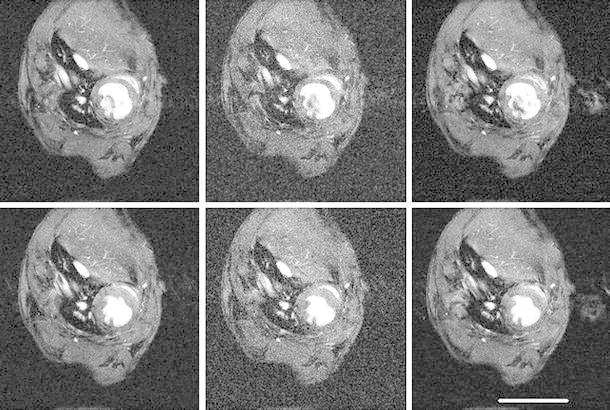
Representative cardiac images of a mouse with myocardial infarction. Mouse imaged 1 week after myocardial infarct. *Upper row* acquired at end diastole and *lower row* at end systole. *Left to right* standard cine sequence with three signal averages (CINE-3); cine sequence with one signal average (CINE-1); kt-BLAST reconstruction of a threefold undersampled sequence (ktB-3). The ejection fraction is noticeably smaller than for the healthy mouse in Fig. 3. Each image has a FOV of 30 mm. *Scale bar* 10 mm

**Fig. 5:**
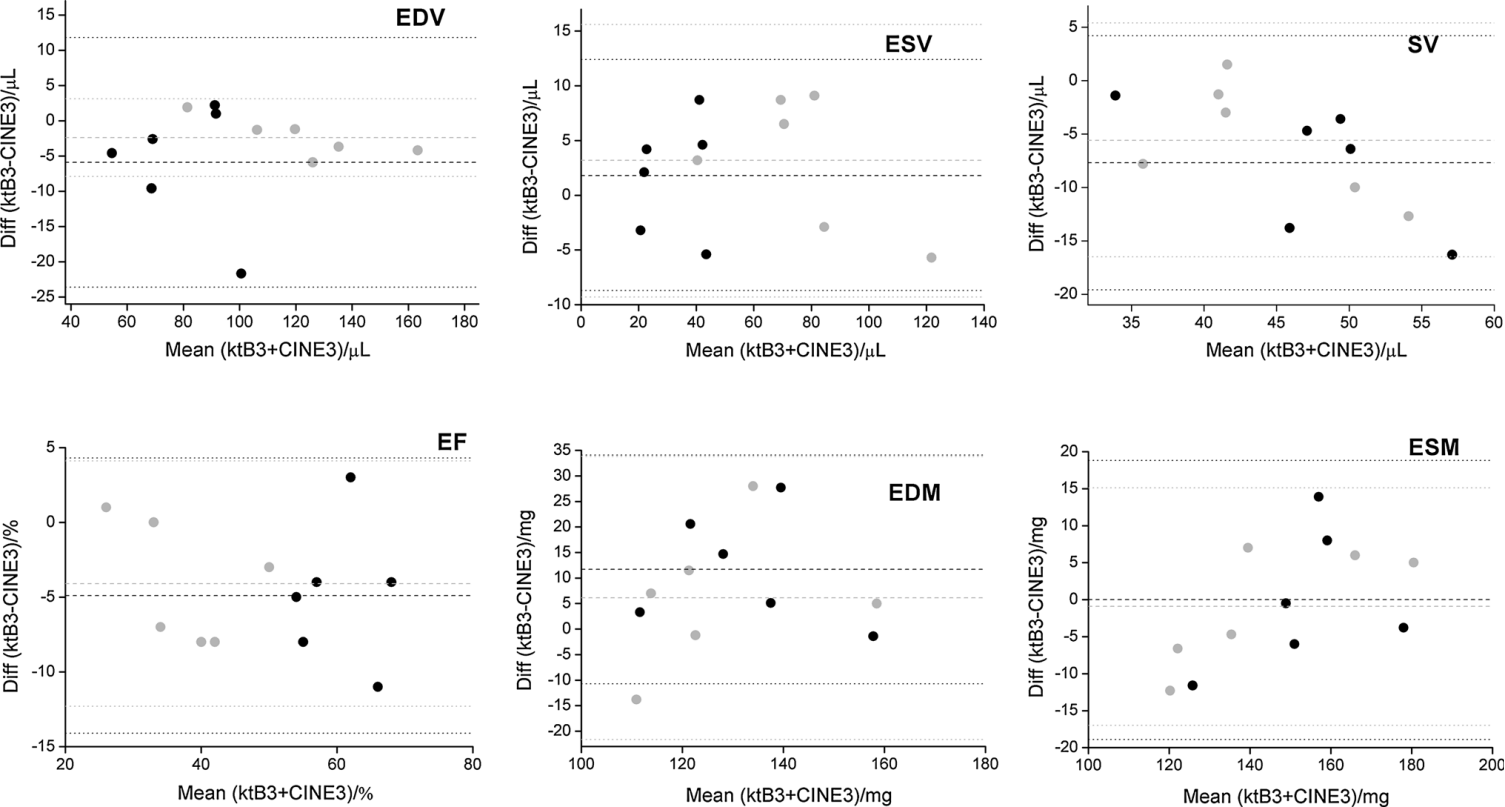
Bland–Altman plots of cardiological indices of left ventricular function [*n* = 6 healthy mice and 6 mice with myocardial infarct (*grey symbols*)], comparing measurements made from threefold kt-BLAST accelerated images (ktB-3) and standard cine images acquired with three signal averages (CINE-3). *EDV* end diastolic volume, *ESV* end systolic volume, *SV* stroke volume, *EF* ejection fraction, *EDM* end diastolic mass, *ESM* end systolic mass. *Dashed lines* indicate bias and *dotted lines* indicate ±2 SD. See also Table 2

**Fig. 6:**
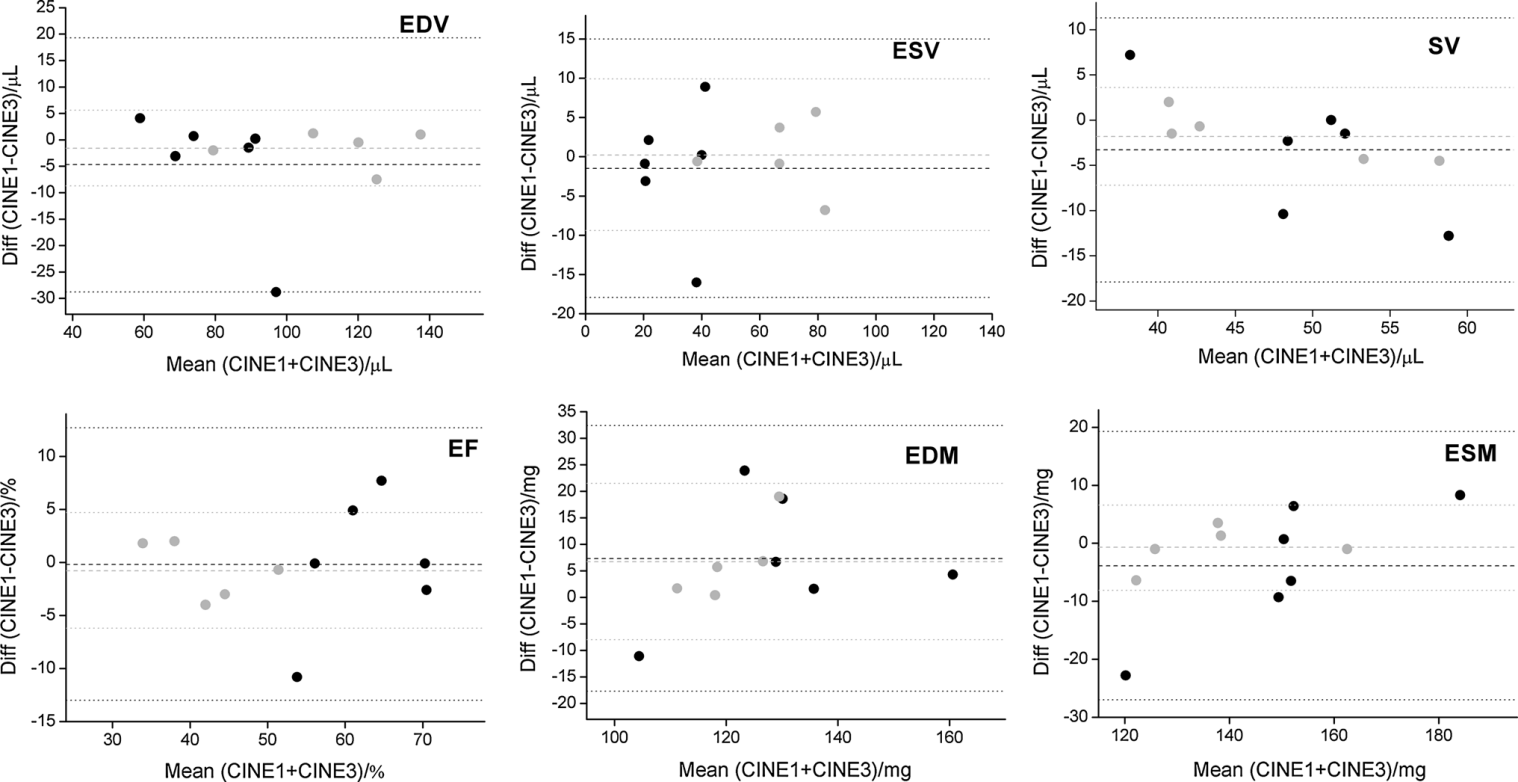
Bland–Altman plots of cardiological indices of left ventricular function [*n* = 6 healthy mice and 5 mice with myocardial infarct (*grey symbols*)], comparing measurements made from cine images with one signal average (CINE-1) and standard cine images acquired with three signal averages (CINE-3). *EDV* end diastolic volume, *ESV* end systolic volume, *SV* stroke volume, *EF* ejection fraction, *EDM* end diastolic mass, *ESM* end systolic mass. *Dashed lines* indicate bias and *dotted lines* indicate ±2 SD. See also Table 2

**Fig. 7:**
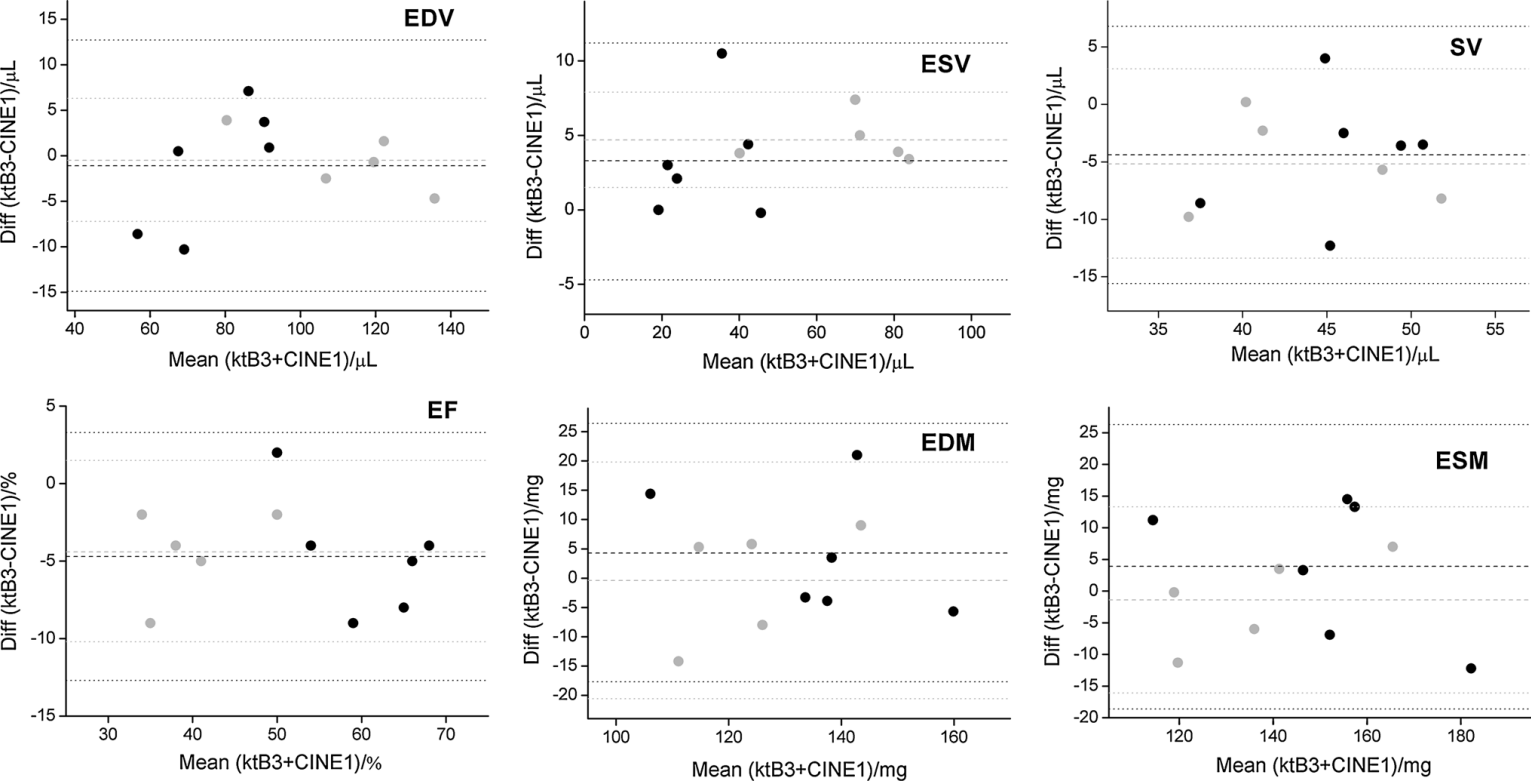
Bland–Altman plots of cardiological indices of left ventricular function [*n* = 6 healthy mice and 5 mice with myocardial infarct (*grey symbols*)], comparing measurements made from threefold kt-BLAST accelerated images (ktB-3) and cine images acquired with one signal average (CINE-1). *EDV* end diastolic volume, *ESV* end systolic volume, *SV* stroke volume, *EF* ejection fraction, *EDM* end diastolic mass, *ESM* end systolic mass. *Dashed lines* indicate bias and *dotted lines* indicate ±2 SD. See also Table 3

Figures 3 and 4 show mid-ventricular images from a healthy and myocardial infarct animal respectively, acquired with the standard (three average), the single-average and the kt-BLAST accelerated scans. The CINE-1 images are noticeably noisier than the others. Table 2 and Figs. 5 and 6 show the group results for the measured cardiological parameters compared to the measurements made from the standard cine sequence. Table 2 also shows the CNR measurements and subjective quality ratings for these scans, which were significantly different from the standard sequence only for the CINE-1 sequence applied to healthy mice. As in the simulations, kt-BLAST acceleration led to overestimation of ESV and underestimation of SV and EF, but the differences did not reach significance. Biases were generally smaller for the CINE-1 sequence than the kt-BLAST sequence. A direct comparison between CINE-1 and kt-BLAST results (Table 3; Fig. 7) revealed no significant differences.

**Table 2:** Accelerated imaging results

	Scan	CNR	Quality (Q)	EDV bias (μL)	ESV bias (μL)	SV bias (μL)	EF bias (%)	EDM bias (mg)	ESM bias (mg)
Healthy	CINE-3	3.4 ± 0.8	4.0 ± 1.3	–	–	–	–	–	–
	CINE-1	2.9 ± 0.6	2.3 ± 1.2*	−4.7 ± 12.0	−1.5 ± 8.2	−3.3 ± 7.3	−0.2 ± 6.4	7.3 ± 12.5	−3.9 ± 11.6
	ktB-3	3.0 ± 0.7	3.5 ± 1.5	−5.9 ± 8.8	1.8 ± 5.3	−7.7 ± 6.0	−4.9 ± 4.6	11.7 ± 11.2	0.0 ± 9.4
Infarct	CINE-3	4.9 ± 1.1	4.5 ± 0.8	–	–	–	–	–	–
	CINE-1	4.6 ± 1.0	3.6 ± 0.9	−1.6 ± 3.6	0.2 ± 4.8	−1.8 ± 2.7	−0.8 ± 2.7	6.7 ± 7.4	−0.7 ± 3.7
	ktB-3	3.6 ± 0.6*	4.0 ± 0.6	−2.4 ± 2.8	3.2 ± 6.2	−5.6 ± 5.5	−4.1 ± 4.1	6.1 ± 13.9	−0.9 ± 8.0

Blood-to-myocardium CNR, subjective image quality rating (Q) and bias from Bland–Altman analysis of cardiological indices of left ventricular function (mean ± standard deviation; *n* = 6 healthy mice plus 5 or 6 mice with myocardial infarct) measured from cine images acquired with one signal average (CINE-1; 5 MI mice) and threefold kt-BLAST acceleration (ktB-3; 6 MI mice), compared to the values measured from standard cine images acquired with three signal averages (CINE-3; 6 MI mice). The range of Q is from 1 (“poor”) through to 5 (“good”). *EDV* end diastolic volume, *ESV* end systolic volume, *SV* stroke volume, *EF* ejection fraction, *EDM* end diastolic mass, *ESM* end systolic mass. * Significantly different (*p* < 0.05) from the corresponding CINE-3 values. See also Figs. 5 and 6

**Table 3:** Comparison between ktB-3 and CINE-1

	EDV bias (μL)	ESV bias (μL)	SV bias (μL)	EF bias (%)	EDM bias (mg)	ESM bias (mg)
Healthy	−1.1 ± 6.9	3.3 ± 4.0	−4.4 ± 5.6	−4.7 ± 4.0	4.3 ± 11.0	3.9 ± 11.2
Infarct	−0.5 ± 3.4	4.7 ± 1.6	−5.2 ± 4.1	−4.4 ± 2.9	−0.4 ± 10.1	−1.4 ± 7.3

Bias from Bland–Altman analysis of cardiological indices of left ventricular function (mean ± standard deviation; *n* = 6 healthy mice plus 5 mice with myocardial infarct) measured from cine images acquired with threefold kt-BLAST acceleration (ktB-3) compared with images acquired with one signal average (CINE-1). *EDV* end diastolic volume, *ESV* end systolic volume, *SV* stroke volume, *EF* ejection fraction, *EDM* end diastolic mass, *ESM* end systolic mass. See also Fig. 7

## Discussion

In this study, we implemented threefold kt-BLAST accelerated cardiac imaging in mice, comparing it with a single-average cine sequence and our standard three-average cine sequence. Images of sufficient quality for analysis were obtained in healthy and infarcted mice.

Initial simulations of kt-BLAST undersampling showed, as expected, that the numerical reconstruction error *ε* increased with increasing simulated acceleration factor *r*. The error manifested itself as increased ghosting artefacts in the reconstructed images, which became more evident at *r* = 4 and *r* = 6. Difference images (such as Fig. 1c, d) clearly showed the artefacts. Estimates of EDV remained acceptably accurate for acceleration factors up to 6, whereas ESV was increasingly overestimated, and hence EF and SV were underestimated. Possible reasons for the overestimation of ESV include more flow artefacts during systole, the smaller heart dimensions than at EDV making papillary muscles harder to define, and reconstruction artefacts taking up a greater proportion of the cardiac cross sectional areas. The errors for *r* = 3 were considered acceptable in the context of reported intra- and inter-observer variabilities in the range 3–11 % [18, 19], and hence this was the target acceleration factor for the subsequent implementation of kt-BLAST. Estimates of left ventricular mass (EDM and ESM) were relatively robust to the simulated acceleration factor.

The differences (group biases) in cardiological parameters between the standard and implemented accelerated acquisitions were of the order of 5–10 %, which are rather higher than the values of approximately 3 % found by Jahnke et al. [11] for left ventricular volumes in a human study comparing standard and kt-BLAST techniques. Many of the reasons discussed above for estimation of ESV may apply, together with the overall lower SNR and CNR of small animal imaging compared with clinical imaging. It might have been expected that true accelerated scans, with training data collected in a separate scan, would have performed worse than simulated versions for which all the data was collected in a single scan. However, no such systematic differences were evident. Parameter biases were generally smaller for the infarcted animals than the healthy animals. In part this was due to the mouse that had the greatest (negative) bias for EDV, ESV and SV (Figs. 5, 6). The reasons for this bias are not entirely clear. Although the CINE-1 images (quality score 3) showed more motion ghosting artefacts than the CINE-3 images (quality score 4), the corresponding kt-BLAST images were not so affected (quality score also 4).

The subjective image quality score Q decreased as the simulated acceleration factor increased, as might be expected. Even for fully sampled k-space, the mean value was 4.22 rather than the theoretical maximum of 5, because of poor quality images in two mice. For the actual kt-BLAST scans, Q was similar to (although slightly higher than) that for the simulated threefold acceleration. Q for the CINE-1 scans was lower, especially for the healthy mice, reflecting the noisy appearance of the images. True blinding of the observer was impossible because of these differences. The contrast between myocardium and left ventricular blood signal intensities is important for segmentation. Measured CNR and Q values were higher in the infarcted hearts than in the healthy hearts, a finding which we ascribe partly to the reduced motion, and correspondingly less motion artefacts, in the infarcted hearts. A simple measure of myocardial motion can be derived from the change in equivalent radius between EDV and ESV. When multiplied by heart-rate this in turns gives a measure of wall velocity. Despite the slightly higher heart-rate of the MI group, the “wall velocity” was significantly less than for the control animals (8.9 ± 2.1 vs 12.7 ± 1.3 mm/s; *p* = 0.01), and may well have contributed to the higher image quality. We used a 39 mm RF coil for imaging the healthy animals since we wanted to explore a wide range of body weights (28–55 g). On the other hand, our method for generation of myocardial infarcts [17] is better suited to younger animals which had lower body weights (22–30 g) and for which we were able to use a 33 mm RF coil. This may also have reduced the noise and hence contributed to increased CNR and Q in the infarcted animals. Since each animal was scanned in the same coil for all three techniques (CINE-3, CINE-1 and ktB3), the comparisons are valid regardless of the exact coil used. As improved coils become available, all the results should improve, but we would expect the relative merits of the three techniques to remain similar.

Acquiring images using the standard cine sequence but with only one signal average, and with the threefold accelerated kt-BLAST sequence allowed a direct comparison between these two approaches to reducing the standard scan time by a factor of three. Visually, acquiring only one average resulted in noisy images, whereas the threefold kt-BLAST acceleration resulted in images that appeared very similar to those acquired with the standard acquisition (Fig. 1). The kt-BLAST reconstruction uses a low-pass filter on the training data, and this promotes smoothness in the resulting images, thereby reducing noise effects at the expense of possible blurring of myocardial edges. Despite their noisy appearance, CINE-1 images gave results slightly nearer to the “gold standard” of CINE-3 images than did the kt-BLAST accelerated images, except for ESM in healthy mice and EDM in infarcted mice. Manual segmentation of the left ventricle thus appears robust to noise at this level. Apart from signal-to-noise considerations, a possible advantage of collecting multiple acquisitions in the presence of periodic and quasi-periodic motion is to reduce artefacts caused by occasional fluctuations in the cardiac or respiratory rates. Even when using dual gating with dummy excitation pulses during respiration so as to maintain the equilibrium magnetisation [20], fluctuations in rates, or incorrect triggering, will cause ghosting artefacts that can generally be reduced by multiple acquisitions. Hence for the same total scanning time, three signal averages with threefold undersampling of k-space might be expected to perform better than a single conventional acquisition in the presence of asynchronous effects. However, in this study, we found that there were no significant differences between the techniques in terms of manual measurement of cardiological indices. The appearance of the CINE-1 images could be improved by “kt-denoising”, that is, exploiting the denoising properties of the kt-BLAST algorithm even in the absence of acceleration [21].

The overall acceleration factor as implemented was actually 2.4 times when taking into account the acquisition of 16 rows of k-space “training” data. It is possible that fewer rows would have sufficed, as reported by others [22], in which case an overall factor nearer to three could have been achieved.

We found no significant biases between cardiological indices derived from kt-BLAST or CINE-1 scans compared with fully sampled scans for healthy mice or for mice with myocardial infarcts. The acceleration methods will therefore be applicable in future studies of haemodynamically compromised mice.

The method of k-space undersampling coupled with kt-BLAST reconstruction can be used with any RF coil setup, and does not require multiple independent receiver coils as does parallel imaging. Undersampling schemes employing compressed sensing reconstruction [12] are also likely to prove useful in rodent studies, and although not specifically optimised for periodic data as is kt-BLAST, the achievable acceleration factors appear similar. Future work should include direct comparisons. Acceleration factors so far reported using parallel imaging in rodent studies are up to four for rats using a four-element coil array [14] and three for mice using an eight-element coil [15]. The combination of parallel imaging and k-space undersampling techniques is expected to lead to further reduction in scanning times, thus improving animal welfare and increasing throughput. Due to use of quadrature coils in the present work, parallel imaging could not be explored or combined with the investigated approach. Acceleration will ultimately be limited by the SNR achievable in rodent imaging studies. For large-scale imaging studies an alternative way of increasing throughput is to scan multiple animals simultaneously [23]. Providing that the difficulties of handling a number of animals simultaneously can be overcome, this method can lead to a significant reduction in the time taken to run a complete study, but does not benefit individual animals.

## Conclusion

Simulations and actual implementation showed that standard cardiological indices in mice can be determined with acceptable accuracy for modest acceleration factors. In this pilot study, running the standard cine sequence with only one average gave slightly (though not significantly) better results (i.e., lower biases) than using threefold kt-BLAST acceleration, even though the image quality was subjectively better for kt-BLAST acceleration. The methods were also shown to be applicable to mice with myocardial infarcts. We have therefore demonstrated that accelerated cardiac imaging of mice is feasible, enabling significant reduction in imaging times.
